# Long-term migration of monoblock vs modular design in uncemented total knee arthroplasty: a secondary report of a randomized trial using radiostereometric analysis

**DOI:** 10.2340/17453674.2025.43904

**Published:** 2025-06-27

**Authors:** Mikkel Rathsach ANDERSEN, Müjgan YILMAZ, Nikolaj WINTHER, Thomas LIND, Henrik SCHRØDER, Gunnar FLIVIK, Michael MØRK PETERSEN

**Affiliations:** 1 Department of Orthopedics, Herlev Gentofte Hospital, University of Copenhagen, Denmark; 2 Department of Orthopedics, Rigshospitalet, University of Copenhagen, Denmark; 3 Department of Orthopedics, Skåne University Hospital, Lund University, Sweden

## Abstract

**Background and purpose:**

Backside wear of the polyethylene insert in total knee arthroplasty (TKA) has been described to produce clinically significant levels of polyethylene debris, which can lead to aseptic loosening and osteolysis. Monoblock design eliminates backside wear of the polyethylene and therefore could improve long-term fixation. This randomized clinical trial (RCT) using radiostereometric analysis (RSA) compares micromotion of monoblock and modular polyethylene inserts with 7 years’ follow-up.

**Methods:**

65 patients (mean age 61 years) were randomized to receive either monoblock (n = 32) or modular (n = 33) uncemented trabecular metal tibial components. 35 patients (monoblock = 18, and modular = 17) completed 7 years’ follow-up. The primary endpoint of the study was maximum total point motion (MTPM). Implant translation and rotation are reported as secondary endpoints.

**Results:**

After 84 months, the modular group had a statistically significant higher mean MTPM of 1.17 (95% confidence interval [CI] 0.90–1.41) mm compared with the monoblock group of 0.78 (CI 0.55–0.88) mm (P = 0.02). However, there was no difference in continuous migration (from 12–84 months), which was 0.13 mm in the monoblock group and 0.16 mm in the modular group.

**Conclusion:**

There was significantly lower early migration in the monoblock group compared with the modular group but no difference in continuous migration after 12 months, which confirms the finding of previous publications.

Aseptic loosening of tibial components in total knee arthroplasty (TKA) is still one of the most common reasons for knee revision [1,2]. Polyethylene debris, with time, can lead to periprosthetic osteolysis and aseptic loosening of tibial implants [3-5]. Backside wear of the polyethylene insert can produce clinically significant levels of polyethylene debris [5,6]. Monoblock design eliminates the backside wear of the polyethylene. Aseptic loosening through particle reactions from polyethylene debris would be expected to occur slowly after a longer period, underlining the importance of long-term follow up to investigate this problem.

An additional theoretical advantage of monoblock design is a more flexible construct than the modular design as there is no titanium plate molded on top of the trabecular metal (TM). This results in a lower modulus of elasticity closer to that of the tibial host bone, which theoretically improves the load-sharing properties of the monoblock implant (Figure 1).

**Figure 1:**
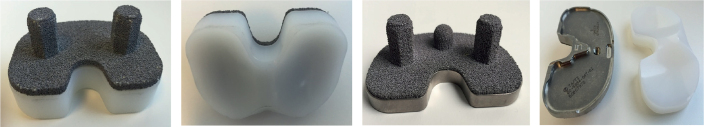
Monoblock (left) and modular (right) trabecular metal surfaced NexGen tibial components (Zimmer-Biomet).

Radiostereometric analysis (RSA) is an efficient method for predicting aseptic loosening of arthroplasty components; high degrees of early migration and continuous migration are both predictors of component loosening. However, for uncemented implants relatively high initial migration is to be expected [7-12].

The aim of our RCT was to investigate the migration of uncemented tibial components of either monoblock or modular polyethylene insert design in a younger group of patients. We hypothesized that the monoblock design would have superior long-term fixation with lower continuous migration than the modular component due to the theoretical design advantages and given the very low migration rates of the monoblock component reported in previous RSA studies [9,10].

## Methods

### Study design

This is a secondary report of an earlier published trial on 2 year results [13].

The study is reported according to CONSORT guidelines.

Patients included in this RCT were recruited and operated on at the Clinic For Hip and Knee Arthroplasty, Gentofte Hospital, Denmark, in the period from August 6, 2012 to the April 25, 2013. 75 patients under the age of 70 years at the time of inclusion and scheduled for uncemented cruciate retaining TKA were included. The patients were randomized to receive the monoblock or modular polyethylene design version of the Cruciate Retaining Trabecular Metal Technology NexGen (Zimmer-Biomet, Warsaw, IN, USA) tibial component. This uncemented tibial component is designed with trabecular metal technology (TMT) made of tantalum with an interconnected pore structure to support biological fixation and vascularization. The polyethylene inserts in both components are highly crosslinked. In the modular group a titanium plate is molded onto the TM to create a locking mechanism; in the monoblock group the polyethylene is molded directly onto the TM. All surgeries were performed by 4 experienced high-volume surgeons, number of surgeries ranging from 11–21 per surgeon.

Randomization (block randomization with 12 in each block) was performed in the operating theatre by opening a closed envelope just prior to the skin incision.

### Radiostereometric analysis

The trial was conducted in accordance with the Guidelines for Standardization of Radiostereometry [14]. The RSA analyses were done using marker-based software (UmRSA v6.0, RSA Biomedical, Umeå, Sweden). Tantalum markers (0.8 mm) were inserted in the tibial host bone and in the polyethylene (1 mm) of the prosthesis during surgery, a method previously validated in several studies [8,10,15]. Markers were placed to create the largest possible nonlinear segments. To ensure comparable maximum total point motion (MTPM) and translational data between the 2 groups, the tantalum beads were placed in a corresponding pattern in both types of implants. 6 markers were placed systematically by the same person for all operations; 2 markers were placed posteriorly, 2 markers were placed at the most medial/lateral part of the polyethylene curve, and 2 markers were placed anteriorly.

The first RSA follow-up was performed after weightbearing with a mean (SD) of 5.0 (2.5) days and 4.9 (2.2) days in the monoblock and modular groups respectively and the 84 months’ follow-up after 83.8 (SD 0.5) and 83.9 (SD 0.5) months from the date of surgery. Patients were positioned in a standardized supine position placing the investigated knee in a plexiglass biplane calibration cage (Calibration Cage 21, Tilly Medical Products, Lund, Sweden), with the coordinate system oriented with the X-axis pointing medially, the Y-axis pointing cranially, and the Z-axis pointing anteriorly. The same physician positioned the patients at each examination. Ceiling-mounted moveable X-ray tubes were positioned perpendicularly in the anterior–posterior and medial–lateral planes at 100 cm distance from the X-ray films placed in portable cassettes. The radio intensity was set at 50 kV and 25 milliampere seconds (mAs) in all examinations. All radiographs were approved by the same physician to ensure sufficient quality. radiographs were digital with 9 pixels per mm. The radiographs were imported to the UmRSA software using the DICOM link v3.0 software (RSA Biomedical, Umeå, Sweden) allowing a resolution of 254 dots per inch.

The distribution of the markers in the rigid bodies is estimated by the software and expressed through the condition number (CN) and the stability of the markers as the mean error (ME). A high CN indicates a narrow and linear distribution of tantalum markers, whereas a low CN indicates good spatial and nonlinear marker distribution. If a marker loosens between examinations the 3D structure of the rigid body deforms, resulting in loss of precision in migration measurements and an increase in ME. The UmRSA software therefore allows loose markers to be removed from a rigid body if the ME becomes too high; however, this will increase CN as spatial distribution of the markers is typically reduced when removing a marker.

To reassure the reliability of the migration results, the CN cut-off in this study was set at 130 and ME cut-off was set at 0.30 mm. We performed 49 double measurements to calculate the precision error for RSA measurements of migration in the study. The patients were asked to step off the bearing and to wait 10 minutes before the second set of radiographs were obtained. We calculated precision error as being 2 standard deviations (2 SD) from a series of theoretical migrations (translations and rotations along all 3 axes, and MTPM) obtained from the 49 pairs of double RSA radiographs, presented in the first publication [13]. Consistent marker method RSA analysis was used.

The migration data in this study are presented as MTPM and segment motion as rotational and translational motion along and around the X-, Y-, and Z-axis. MTPM was calculated from the marker with largest translational vector; no fictive points were used. Translations were measured at a centroid polygon from the 6 markers placed systematically in the insert, and the number of markers was controlled at each examination in order to ensure no markers were missing. The results of rotations and translations are presented as signed values.

### Ethics, data sharing plan, funding, use of AI, and disclosures

The study was approved by the Scientific Ethical Committee of Copenhagen (H-1-2012-033), and conducted in accordance with the Helsinki declaration with informed consent obtained (after written and oral information) from all study participants prior to inclusion in the study. The study was approved by the Danish Data Protection Agency (ID 01766, GEH-2012-027), and the study was registered at ClinicalTrials.gov (NCT01637051) prior to study start. Patient-level data is available on request for sharing with researchers after the study’s completion in SPSS format for research use only and in agreement with Danish General Data Protection Regulation law. Data is available by contacting the corresponding author. There has been no use of AI in conducting this study or in manuscript writing. The study received an institutional research grant from Zimmer Biomet. No competing author disclosure was reported. Complete disclosure of interest forms according to ICMJE are available on the article page, doi: 10.2340/17453674.2025.43904

### Statistics

This is a confirmatory trial, and the primary endpoint of the study was to compare the migration expressed as MTPM of the 2 tibial component designs at each follow-up during the 84 months’ follow-up period. MCID was for MTPM was 0.2 mm [15]. In the first publication of this cohort, sample size calculation was presented based on MTPM after 24 months as the primary outcome parameter [13]. The segment motion (translations and rotations) data are considered secondary explanatory endpoints.

The migration data (MTPM and segment motions) in both groups was not normally distributed. We used a Mann–Whitney U-test for testing of differences in MTPM between the 2 groups.

Statistics were compiled using SPSS statistics v. 21 (IBM Corp, Armonk, NY, USA) and data is presented as mean values together with total range, standard deviation (SD; for graphical presentations only), or 95% confidence interval (CI). P values below 0.05 were considered significant. Sample size calculation is presented in the first publication [13].

## Results

**Figure 2:**
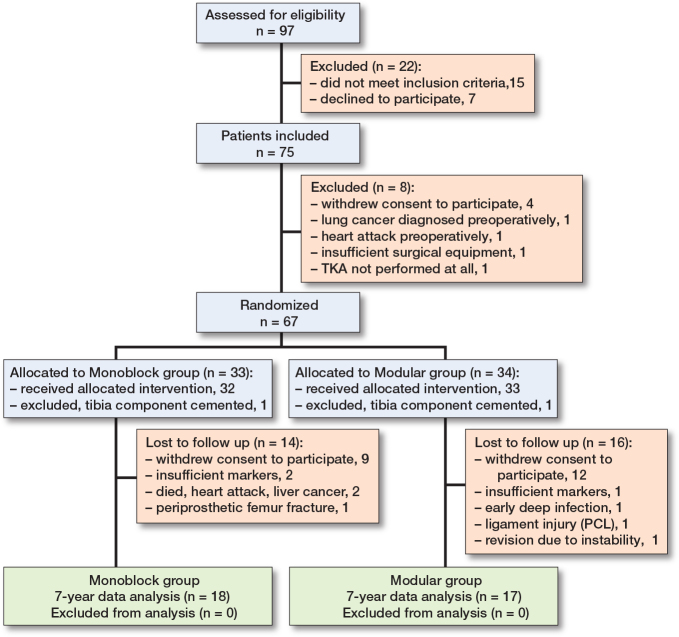
Flowchart of study.

Of the 75 patients included in the study, 8 were excluded before randomization because the patient withdrew his/her consent to participate in the study (n = 4), or surgery was not performed (n = 4) (Figure 2). In 2 cases, the surgeon decided to cement the tibial component after randomization (n = 2). Thus, 65 patients (mean age 61 years; 37 women and 28 men) received the allocated intervention, with 32 in the monoblock group and 33 in the modular group. Since the previously published 2-year follow-up [11] there had been no further revisions. During the follow-up period 3 were revised, 1 in the monoblock group due to periprosthetic femur fracture (MTPM at 12 months 0.7 mm), and 2 in the modular group, 1 due to early infection (MTPM at 4 days, 0 mm) and 1 due to instability (MTPM at 12 months, 1.0 mm).

35 patients (monoblock = 18, and modular = 17) completed 7 years’ follow-up and full data analysis (Figure 2).

The groups were comparable with regards to preoperative demographics (Table 1).

**Table 1:** Patient demographics, preoperative demographics for allocated groups, and groups with completed 84 months’ follow-up. Values are mean (range) or count

Parameters	Allocated to Monoblock (n = 32)	Allocated to Modular (n = 33)	At 84 months Monoblock (n = 18)	At 84 months Modular (n =1 7)
Sex (F/M), n	18/14	19/14	10/8	9/8
Age (years)	62 (47–70)	61 (48–70)	74 (63–83)	73 (61–83)
Body mass index	29 (22–40)	29 (24–40)	28 (22–38)	28 (24–40)
Valgus knees, n	10	7	6	5
Varus knees, n	22	26	12	12
Ahlbäck score	3.0 (2–4)	3.2 (2–5)	–	–

Ahlbäcks classification of knee osteoarthritis:

1: joint space narrowing (<3 mm)

2: joint space obliteration

3: minor bone attrition (0–5 mm)

4: moderate bone attrition (5–10 mm)

5: severe bone attrition (>10 mm)

### Outcomes

**Figure 3:**
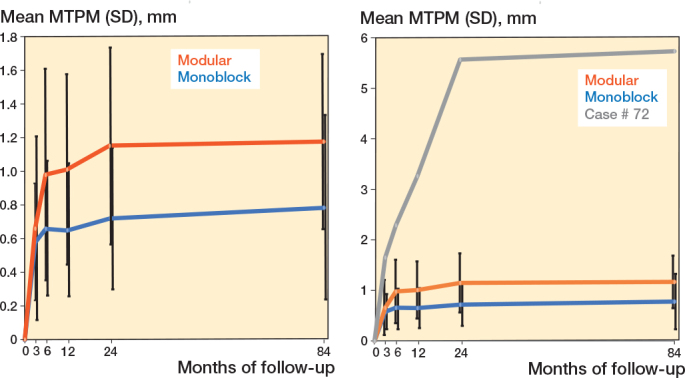
(Left panel) Mean maximum total point motion (MTPM), mm (SD), 0–84 months. (Right panel) MTPM for outlier case # 72.

We found the largest change in mean MTPM within the first 6 months in both the modular group and the monoblock group reaching 0.98 (CI 0.74–1.26) mm and 0.66 (CI 0.44–0.77) mm respectively (Table 2, Figure 3).

**Table 2:** Group migration data up to 84 months’ follow-up. Values are mean (95% confidence interval)

Item	3 months	6 months	12 months	24 months	84 months
Monoblock, n	32	31	30	26	18
Modular, n	32	32	32	27	17
Maximum total point motion, mm					
Monoblock	0.58 (0.43 to 0.72)	0.66 (0.44 to 0.77)	0.65 (0.46 to 0.78)	0.72 (0.53 to 0.82)	0.78 (0.55 to 0.88)**^a^**
Modular	0.85 (0.65 to 1.07)	0.98 (0.74 to 1.26)	1.01 (0.81 to 1.28)	1.15 (0.90 to 1.37)	1.17 (0.90 to 1.41)**^a^**
Superior (+)/ inferior (–) translation, mm					
Monoblock	–0.16 (–0.22 to –0.09)	–0.17 (–0.23 to –0.08)	–0.18 (–0.23 to –0.06)	–0.18 (–0.24 to –0.07)	–0.13 (–0.22 to –0.07)
Modular	–0.33 (–0.47 to –0.19)	–0.33 (–0.51 to –0.21)	–0.37 (–0.52 to –0.22)	–0.38 (–0.54 to –0.23)	–0.36 (–0.60 to –0.26)
Medial (+)/lateral (–) translation, mm					
Monoblock	0.01 (–0.05 to 0.08)	0.01 (–0.05 to 0.07)	0.02 (–0.01 to 0.10)	–0.02 (–0.09 to 0.01)	–0.04 (–0.20 to 0.13)
Modular	–0.04 (–0.20 to 0.14)	0.00 (–0.09 to 0.08)	–0.01 (–0.08 to 0.07)	–0.03 (–0.09 to 0.08)	–0.04 (–0.21 to 0.12)
Anterior (+)/posterior (–) translation, mm					
Monoblock	–0.15 (–0.25 to –0.04)	–0.15 (–0.27 to –0.00)	–0.19 (–0.37 to –0.01)	–0.15 (–0.36 to –0.06)	–0.15 (–0.35 to –0.05)
Modular	–0.22 (–0.35 to –0.11)	–0.23 (–0.39 to –0.07)	–0.28 (–0.45 to –0.11)	–0.33 (–0.55 to –0.16)	–0.45 (–0.65 to –0.23)
Anterior (+)/posterior (–) tilt, °					
Monoblock	–0.51 (–0.73 to –0.29)	–0.52 (–0.74 to –0.26)	–0.58 (–0.8 to –0.29)	–0.53 (–0.8 to –0.22)	–0.62 (–0.91 to –0.09)
Modular	–0.68 (–0.96 to –0.32)	–0.69 (–1.07 to –0.33)	–0.83 (–1.17 to –0.40)	–0.92 (–1.35 to –0.48)	–1.15 (–1.52 to –0.60)
Varus (+)/valgus (–) tilt, °					
Monoblock	0.06 (–0.04 to 0.25)	0.02 (–0.06 to 0.20)	–0.01 (–0.12 to 0.09)	–0.03 (–0.18 to 0.06)	–0.04 (–0.10 to –0.14)
Modular	0.01 (–0.09 to 0.11)	0.01 (–0.10 to 0.14)	–0.04 (–0.21 to 0.08)	–0.02 (–0.15 to 0.03)	0.07 (0.0 to 0.16)
Internal (+)/external (–) rotation, °					
Monoblock	–0.11 (–0.17 to –0.07)	–0.15 (–0.19 to –0.10)	–0.19 (–0.26 to –0.13)	–0.25 (–0.28 to –0.15)	–0.44 (–0.56 to –0.08)
Modular	–0.09 (–0.16 to 0.04)	–0.14 (–0.26 to 0.07)	–0.14 (–0.21 to –0.08)	–0.16 (–0.25 to –0.08)	–0.18 (–0.26 to –0.14)

a P = 0.02 for Monoblock versus Modular group at 84 months.

Thereafter the MTPM migration curves flattens towards 84 months. The difference in mean MTPM at 84 months between the groups was statistically significant with 1.17 (CI 0.90–1.41) mm in the modular group and 0.78 (CI 0.55–0.88) mm in the monoblock group. In both groups there was little continuous migration, with an average 6–84 months’ MTPM change of 0.12 mm in the monoblock group and 0.19 mm in the modular group and a 12–84 months’ MTPM change of 0.13 mm and 0.16 mm respectively. There was no statistically significant difference between the groups in continuous migration.

There was 1 outlier after 24 months in the monoblock group, case no. 72, with an MTPM value of 5.5 mm. Interestingly, this patient’s tibial implant stabilized with only a little further migration at the 84 months’ follow-up (Figure 3).

**Figure 4:**
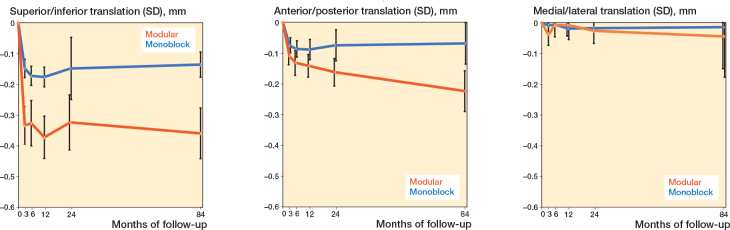
(Left panel) Mean superior-inferior translations, mm (SD). Negative values indicate subsidence. (MIddle panel) Mean anterior–posterior translations, mm (SD). (Right panel) Mean medial–lateral translations, mm (SD).

The implants’ directional migrations along the 3 axes are referred to as translations. We found a relatively large difference in negative Y-translation (subsidence) between the groups at the 84 months’ follow-up, with 0.13 (CI 0.07–0.22) mm and 0.36 (CI 0.26–0.60) mm in the monoblock and modular groups, respectively (Table 2). Most of the subsidence occurred within the first 3 months in both groups (Figure 4, Table 2). We found a negative Z-translation (anterior–posterior axis) in both groups of 0.45 (CI 0.23–0.65) and 0.15 (CI 0.05–0.35) mm in the monoblock and modular groups (Figure 4). There was very little X-translation (medial–lateral axis) in either group (Figure 4).

The largest rotational migration was a negative X-rotation (posterior tilt) in both groups. The monoblock implants rotated an average of 0.62° (CI 0.09–0.91) and the modular group 1.15° (CI 0.60–1.52). The monoblock and modular implants rotated 0.44° (CI 0.08–0.56) and 0.18° (CI 0.14–0.26) externally, respectively (negative Y-rotation). There was very little varus/valgus tilt (Z-axis rotation) of the components in either group. The PE, ME, and CN are presented in the first publication [13].

From 49 double examinations we calculated an ME of 0.15 mm for the tibial bone segment and 0.07 mm for the implant segments.

There were no further revisions after the 84 months’ follow-up. Clinical outcome scores were good in both groups at the 24 months’ follow-up with KSS function score of 94 (60–100) in the monoblock groups and 91 (50–100) in the modular group [13]. Clinical scores were not reported at the 84 months’ follow-up.

## Discussion

This is the first RSA study comparing monoblock and modular tibial components with uncemented fixation of both components. We aimed to compare the migration of uncemented tibial components of either monoblock or modular polyethylene insert design in a younger group of patients and we found a higher migration in the modular components after 84 months, but there was no difference in the long-term continuous component migration. We therefore reject our hypothesis that the monoblock component would achieve superior long-term fixation due to elimination of backside polyethylene wear and a lower modulus of elasticity of the tibial baseplate construction. Publishing the long-term migration data of this trial, we believe is essential to evaluate the hypothesis of this trial, as a potential polyethylene-debris-induced difference in fixation would be expected to develop over a long period of time. Regarding the significant difference in initial migration between the groups, we have no scientific grounds to contribute this to the difference in modulus of elasticity and load-sharing properties, and we found no clinical relevance of the difference in the study population.

High initial migration is to be expected in uncemented tibial components and the initial migration of both components is within previously published values [7-10,16,17].

With the introduction of highly-crosslinked polyethylene inserts, aseptic loosening due to polyethylene debris could become a decreasing cause for revision knee arthroplasty. A meta-analysis including 961,467 primary total knee arthroplasties recommends the routine use of highly crosslinked liners in TKA because of a significantly lower revision rate due to aseptic loosening [21].

A recently published metanalysis compared monoblock with modular design and presented RSA data, complications, revision rates, and clinical outcome scores, from 7 RCTs with a total of 635 cases, including the 24 months’ data of the 53 cases from this study [13,22]. The metanalysis included 5 studies with RSA migration data. However, the modular components used in the other 4 studies were cemented, which means that the patients were randomized to cemented versus uncemented fixation. We believe that such comparison will give little knowledge regarding the significance of monoblock versus modular design, and these trials are primarily a comparison of cemented versus uncemented fixation, as the use of cemented fixation will have a much larger effect on the migration pattern than the modularity of the component. Regarding the use of uncemented fixation for tibial components, the metanalysis found significantly lower migration in the uncemented monoblock group at 2-year follow-up when compared with cemented fixation. The metanalysis also reports a significantly better outcome score in the uncemented monoblock groups, whereas revisions and complications were equivalent [22].

In previous RSA studies the uncemented monoblock component has shown excellent early and long-term migration results in a younger population [9,16]. The data from our trial confirms the previously published data on the monoblock component. We did, however, find significantly higher early mean migration in the modular group, indicating inferior initial fixation. RSA meta-analysis has shown that, when looking at larger groups, early mean component migration is important and associated with a higher risk of aseptic loosening on a group level, whereas mean continuous migration is not [7,17]. In a study with few cases like ours, an association between early large migration and aseptic loosening could be overlooked due to a low number of cases in each group.

### Limitations

The primary limitation of this study is the number of dropouts during the follow-up period from 24–84 months. 18 patients dropped out, and only 35 patients completed 84 months of follow-up (see Figure 2) which is significantly less than the calculated sample size. This increases the risk of false negative results (Type II error) and could potentially mask a difference in continuous migration between the groups. There is the potential for dropout bias to exist, i.e., patients with poor outcome, old age, or possibly high migration dropping out; however, when comparing the demographics for the groups that completed 84 months’ follow-up, we find no such indication (see Table 1). We also compared the early migration data of the dropout group compared with the group with a full 84 months of follow-up, in order to assess the risk of dropout bias, and found no indication for a higher degree of migration in the dropout group (see Table 3). We consider the dropout group to be random, and a result of inconvenience and other factors not related to the TKA.

**Table 3:** Mean MTPM for dropout patients and patients with completed follow-up, 0–24 months. Values are mean (CI) MTPM, mm and [number of cases]

Follow up	3 months	6 months	12 months	24 months
Complete	0.60 [64]	0.84 [63]	0.87 [62]	0.92 [53]
CI	0.43–0.78	0.59–1.12	0.66–1.10	0.81–1.30
Dropout patients	0.65 [1]	0.88 [2]	0.90 [3]	1.03 [12]

CI: 95% confidence interval

Another possible limitation to this study is that the 84 months’ follow-up period could be a too short a time period to reveal a difference between the groups due to polyethylene-induced loosening. A meta-analysis with a mean follow-up of 6 years found no significant difference in revision between highly crosslinked versus conventional polyethylene in primary TKA [23]. We cannot assume that the difference in amount of polyethylene debris produced between monoblock and modular designs corresponds to that of highly crosslinked and conventional polyethylene, but this could be an indication that a longer follow-up period is needed to show a difference.

A further limitation of this study is that it is not possible to maintain patient and examiner blinding, as the difference in tibia design is visible on radiographs; however, this should not be an issue concerning component migration as the RSA data comprises objectively measured outcomes.

### Conclusion

There was significantly lower early migration in the monoblock group compared with the modular group, which confirms the finding of previous publications.

*In perspective,* knee arthroplasty surgeons’ preference for a modular design in uncemented tibial components is evident in current practice worldwide. However, the low migration of monoblock components documented through RSA studies should be taken into consideration, and weighed against the advantages of modularity.

## Notes

MRA: design, acquisition, analysis, interpretation, writing; MYA: acquisition, analysis; TL: design, acquisition; NW: design; HS: design; GF: design, analysis; MMP: design, acquisition, analysis, interpretation, reviewing.

Håkan Leijon is thanked for his contribution to the RSA analysis.

Handling co-editor: Per Henrik Randsborg

*Acta* thanks Bart G Pijls and Arkan S. Sayed-Noor for help with peer review of this manuscript.
